# Diagnostic value of four-dimensional CT angiography in arterial erectile dysfunction using 320-detector row dynamic volume CT

**DOI:** 10.1042/BSR20170200

**Published:** 2017-08-21

**Authors:** Cheng-Cheng Xu, Xin-Zhong Ruan, Yi-Fan Tang, Jiao-Hai Pan, Guo-Yao Wang, Qiu-Li Huang

**Affiliations:** 1 Department of Imaging, Ningbo First Hospital, Ningbo 315010, P.R. China; 2 Department of Urology, Ningbo First Hospital, Ningbo 315010, P.R. China

**Keywords:** Arterial erectile dysfunction, Diagnosis, 320-row dynamic volume CT, Four-dimensional CT angiography, Specificity, Sensitivity

## Abstract

The present study aims to evaluate the diagnostic value of four-dimensional CT angiography (4D-CTA) in the diagnosis of arterial erectile dysfunction (ED) using 320-detector row dynamic volume CT. Arterial ED patients attributed to arterial insufficiency were enrolled. To induce penile erection, an intracavernous injection (ICI) of corpus cavernosum with a vasoactive drug was administered. Patients were assigned into the erection hardness score (EHS) 1/2 group or EHS 3/4 group. Color duplex Doppler ultrasound (CDDU) was used to analyze blood flow spectrum. Each patient was examined using 4D-CTA. Receiver operating characteristic (ROC) curve was plotted to evaluate the diagnostic value of 4D-CTA in arterial ED. According to Irwin Goldstein, the EHS 3/4 group (*n*=38) had a shorter course of ED and low proportion with history of hypertension, hyperlipidemia, and diabetes than the EHS 1/2 group (*n*=35). The peak systolic velocity (PSV), end diastolic velocity (EDV), and resistant index (RI) in the EHS 3/4 group were lower than those of the EHS 1/2 group. 4D-CTA showed there were a total of 35 cases in the EHS 1/2 group (two cases missed) and 38 cases in the EHS 3/4 group (seven cases misdiagnosed). Using 4D-CTA to diagnose arterial ED, the area under the ROC curve yielded a value of 0.879, with a specificity of 93.9% and a sensitivity of 82.5%. These findings indicated that 4D-CTA using 320-detector row dynamic volume CT is a promising and reliable utility in diagnosing arterial ED.

## Introduction

Erectile dysfunction (ED) is defined as the inability to reach potential or maintain a full erection required for satisfying sexual performance [1]. It has been estimated that the worldwide prevalence of ED will be 322 million cases by the end of 2025 [2]. ED is multifactorial and the causes may include neurogenic disorders, cavernosal disorders (peyronie’s disease), psychological disorders (performance anxiety, stress, and mental disorders), surgery, and aging [3–7]. As for the diagnosis of ED, color duplex Doppler ultrasound (CDDU) is of great significance in identifying the most appropriate therapy [8]. However, poor reproducibility is considered as the main bias of penile artery dynamic Doppler examination, and psychological issues are considered to be mainly responsible for the aforementioned [9]. Furthermore, due to lack of standardized measures, it is difficult to obtain accurate clinical diagnosis of a patient undergoing hemodynamic assessment of the penis by CDDU [8]. In addition, due to the complexity of ED and inadequate availability of studies, there are still many limitations to our understanding of ED [10].

The 320-detector row dynamic volume CT consisting of 320-slice detectors that cover a width range of 16 cm along the *z*-axis, 0.5 m of the thickness, and 350 ms of gantry rotation time, which reliably provides high diagnostic accuracy without heart rate/rhythm control [11]. Making it possible to accurately image the flow of contrast in the cerebral vasculature by conducting consecutive volumetric scans over a predetermined time frame: this type of time-saving CT can be applied to estimate the hemodynamics, as well as the morphology, of various cerebrovascular conditions [12]. Originating in CT perfusion (CT), four-dimensional CT angiography (4D-CTA) has been classified as noninvasive and is better at estimating the collateral status [13]. During the past few decades, 4D-CTA using 320-detector row dynamic volume CT has been the attention of researchers in the diagnosis of diseases such as atherosclerosis causing myocardial ischemia, vascular clips or endovascular coils, and developmental venous anomalies [14–16]. Its characteristic findings have been of great importance in evaluating the disease severity and in monitoring response to treatment [17]. Therefore, the present study was designed to investigate the diagnostic value of 4D-CTA in the diagnosis of arterial ED using 320-detector row dynamic volume CT.

## Materials and methods

### Study subjects

A total of 73 patients (the age range was 19–52 years old with mean age of 30.3 ± 5.8 years, and course of disease ranged from 2 to 96 months) admitted in the Ningbo First Hospital with arterial ED for the first time between the period of March, 2014 to March, 2016 were enrolled for this study. The inclusion criteria were as follows: patients had low or no erection ability as tested by nocturnal penile tumescence (NPT) and audiovisual sexual stimulation (AVSS); arterial ED patients diagnosed by CDDU with peak systolic velocity (PSV) < 35 cm/s (when PSV is lower than 35 cm/sec in young patients, it is mainly due to psychological causes) [18]. Exclusion criteria: patients with traumatic injuries of the penis, spinal cord injury, or long-term use of drugs known to affect sexual function. The study performed with approval of the Ethical Committee of Ningbo First Hospital and the participating patients signed informal consents.

### NTP and AVSS evaluation

NTP: The patients were requested to undergo examination in the examination room alone. The portable RigiScan was put on the patients at 21:00 every night, and were taken out at 8:00 the next day, the examination was conducted for three nights. Next, the data were recorded.

AVSS: The patients were requested to stay in special examination room (quiet and avoiding light), and watch erotic videos for 45 min. The RigiScan was used to record erection hardness, and the data were analyzed [19].

### Clinical history and physical examination

Blood samples were withdrawn from all fasting patients in the morning. Serum biochemistry was used to test blood glucose and lipids. All men had a detailed consultation and physical examination, with special attention to relevant history of masturbation, diabetes, hypertension, hyperlipidemia, and angiocardiopathy. The patients also received evaluations scores for Self-rating Anxiety Scale (SAS) [20] and Self-rating Depression Scale (SDS) [21].

### Inducing erection with intracavernous injection

After intracavernous injection (ICI) for inducing erection, patients were positioned in a supine position. A constriction ring was placed on the root of the penis to prevent venous return. A combination of papaverine (30 mg) and phentolamine (1 mg) was injected into cavernosa after disinfection. After compressing the penis for 3–5 min, the penile deep arteries and cavernosal arteries were bidimensionally observed before and after injecting. According to Irwin Goldstein, there were four erection hardness levels: level I: not hard or no erection; level II: hard but not hard enough for penetration; level III: hard enough for penetration but not completely hard; level IV: completely hard and fully rigid. The four levels were assigned into Erection Hardness Score (EHS) 1/2 group (level I and level II ) and EHS 3/4 group (level III and level IV) [22].

### Color duplex Doppler ultrasound

A CDDU machine (Biosound-AU4, America) with ultrasonic probe frequency of 13 MHz and the color Doppler probe frequency of 7 MHz was used. The ultrasonic transducer was placed on the root of penis. The color Doppler probe was employed to detect blood flow level in the blood vessels after ICI and to measure the perimeter and length of the penis, with the angle of sampling ultrasonic beam and vessel diameter not greater than 60°. Spectrum of blood flow was obtained and analyzed. Vascular examination was performed to record the PSV, end diastolic velocity (EDV), and resistant index (RI).

### Four-dimensional CT angiography

Iodine allergy was tested 30 min before angiography. A combination of papaverine (30 mg) and phentolamine (1 mg) was injected into the cavernosa to induce an erection. After 3–5 min of compression on the injection point, patients were positioned in a supine position and indirect CTA was performed. Hundred milliliters (350 mgI/ml) of nonionic iodinated contrast agent was intravenously injected with a high pressure injector at a flow rate of 4 ml/s. Efforts were made to ensure that the scan coverage was ranging from the internal iliac artery to the root of the penis. Dynamic volume CT scanning was employed via 320-detector row dynamic volume CT (Toshiba Aquilion One; Toshiba Corporation, Kawasaki, Japan) with the following parameters: rotation speed of the ball tube 0.35 s/rot, slice thickness 1.5 mm, coverage area 16 cm, and field of view (FOV) 240 mm. The 65 ml (370 mgI/ml) of nonionic contrast agent iopamidol was intravenously injected at a rate of 5.0 ml/s, followed by 30 ml of normal saline at the same rate. After a 7 s delay post-injection, CT scanning was performed for 50–60 s.

All the images were reconstructed with 0.5 mm slice thickness. Each volume data contained 320 images, and 3200 images were recorded from each examination. Next, the data were imported to four-dimensional digital subtraction angiography (DSA) for acquiring four-dimensional dynamic vascular images. Multiple planar reformation (MPR), maximum intensity projection (MIP), and volume rendering (VR) were performed to visualize the penis. Patients with lesions in internal artery and/or cavernosal arteries, stenosis or no development in the dorsal artery of the penis were assigned to the EHS 1/2 group.

### Statistical analysis

Data were analyzed using the statistical package for the social sciences (SPSS) version 21.0 (SPSS Inc., Chicago, IL, U.S.A.). The *t*-test was used for comparison between two groups of measurement data. The measurement data were recorded as mean ± standard deviation (SD). Measurement data were expressed as percentage and rate, and were analyzed using chi-square test. ROC curve was applied to quantify the diagnostic value of 4D-CTA for arterial ED patients. *P*<0.05 was regarded as statistically significant.

## Results

### Baseline characteristics of arterial ED patients

Among the 73 ICI induced arterial ED patients, level I included 13 patients, level II had 22 patients, and level III had 9 patients, whereas level IV had 29 patients. According to Irwin Goldstein, the EHS 1/2 group contained 35 cases while the EHS 3/4 group consisted of 38 cases. Table 1 shows the detailed baseline characteristics of the subjects. No significant differences were observed in terms of age, anxiety, and depression between the two groups. Notably, the EHS 3/4 group experienced the condition for a shorter duration and had less cases with any prior history of hypertension, hyperlipidemia, diabetes, and masturbation than the EHS 1/2 group (all *P*<0.05).

**Table 1:** Baseline characteristics of 73 arterial ED patients

Characteristic	EHS 1/2 group (*n*=35)	EHS 3/4 group (*n*=38)	*t*/χ^2^	*P*
Age (years)	30.60 ± 6.21	30.03 ± 5.50	0.42	0.679
Course of disease (months)	30.89 ± 16.57	18.95 ± 6.18	4.14	<0.001
Anxiety	11	14	0.24	0.626
Depression	10	12	0.08	0.780
History of hypertension	8	2	4.77	0.029
History of hyperlipidemia	12	5	4.55	0.033
History of diabetes	6	1	4.43	0.035
History of masturbation	9	3	4.21	0.040

### Color duplex Doppler ultrasound scanning of arterial ED patients

**Figure 1:**
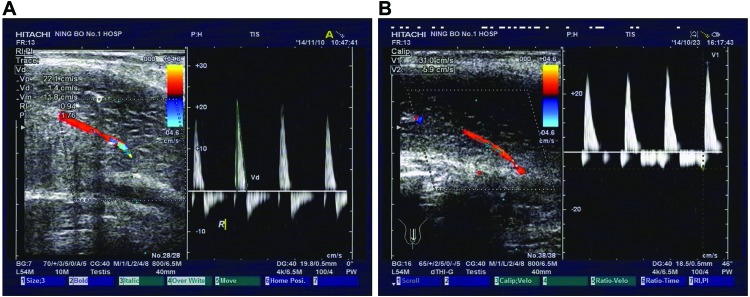
Color duplex Doppler ultrasound scanning of arterial ED patients in the EHS 1/2 (A) and EHS 3/4 (B) groups

Arterial ED patients in the EHS 1/2 group (*n*=35) had a single-peak spectrum in systole with no or a rather low blood flow spectrum in diastole (Figure 1A). Arterial ED patients in the EHS 3/4 group (*n*=38) had a greater blood flow spectrum in both systole and diastole after erection (Figure 1B). The EHS 3/4 group had lower PSV, EDV, and RI than the EHS 1/2 group (all *P*<0.05) (Table 2).

**Table 2:** Comparisons of CDDU parameters between the EHS 1/2 group and the EHS 3/4 group

Parameter	EHS 1/2 group (*n*=35)	EHS 3/4 group (*n*=38)	*t*	*P*
PSV (cm/s)	30.97 ± 5.16	22.81 ± 2.79	8.30	<0.001
EDV (cm/s)	7.94 ± 1.57	3.28 ± 1.02	14.90	<0.001
RI	0.95 ± 0.07	0.72 ± 0.05	16.03	<0.001

### Four-dimensional CT angiography of arterial ED patients

**Figure 2:**
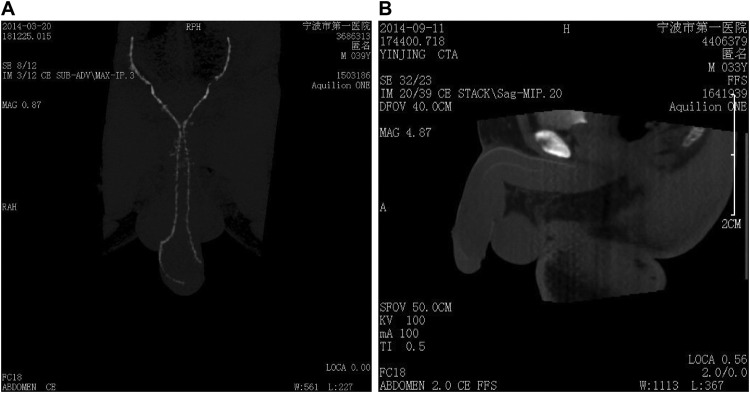
Four-dimensional CT angiography of arterial ED patients in the EHS 1/2 (A) and EHS 3/4 (B) groups

The 4D-CTA showed that among the 35 arterial ED patients in the EHS 1/2 group, two cases were missed diagnosis, and there were nine cases with lesions in internal carotid artery, 14 cases in cavernous artery, five cases in penile dorsal artery, and five cases in internal carotid vein, with obvious stenosis or no development compared with the normal contralateral artery (Figure 2A). Among 38 arterial ED patients in the EHS 3/4 group, there were seven cases of misdiagnosis. The Figure 2(B) showed internal carotid artery, cavernous artery, and penile dorsal artery, with contrast agent filling and no obvious artery stenosis or development. The results proved that 4D-CTA could display the cavernous artery, internal carotid artery, and penile dorsal artery simultaneously, thereby clearly identifying lesion sites.

### Evaluating the diagnostic value of 4D-CTA in patients with arterial ED using ROC curve

**Figure 3:**
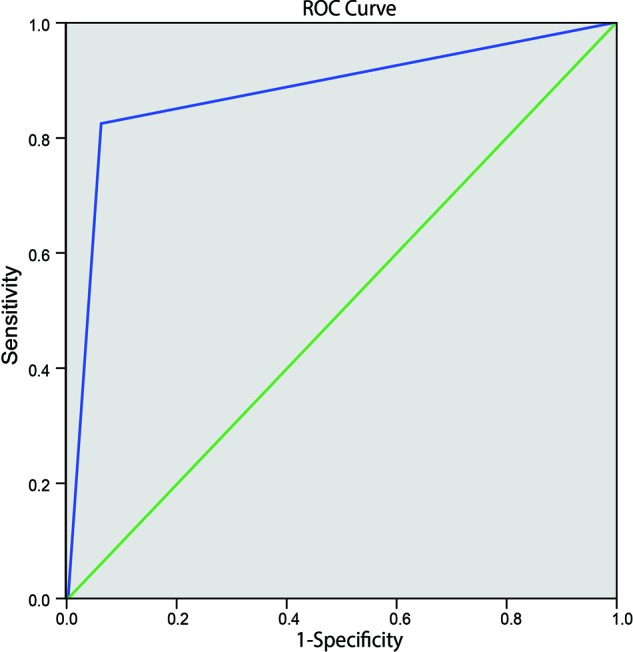
Evaluation of the diagnostic value of 4D-CTA in patients with arterial ED using ROC curve

Compared with the ‘Gold Standard’ of CDDU, area under the ROC curve (AUC) of 4D-CTA for the diagnosis of arterial ED was 0.879, with a specificity of 93.9%, a sensitivity of 82.5%, positive predictive value of 94.3%, negative predictive value of 81.6%, and diagnostic accuracy of 87.7%. The accuracy of 4D-CTA in diagnosing arterial ED was comparable to that of CDDU (Figure 3).

## Discussion

It has been reported that CDDU may aid in the decision-making process regarding choosing the most appropriate and right therapy for treating ED. Unfortunately, there is no uniform standardization in performing CDDU resulting in high variability in data expression and interpretation while comparing results among various centers, especially when conducting multicenter trials [8]. Therefore, the present study aimed to investigate the effectiveness of 4D-CTA in the diagnosis of arterial ED using 320-detector row dynamic volume CT. Our findings revealed that 4D-CTA using 320-detector row dynamic volume CT was a very promising and efficient option for accurately diagnosing arterial ED.

In the present study, CDDU results showed that in the EHS 1/2 group (*n*=35), PSV was 30.97 ± 5.16, EDV 7.94 ± 1.57, and RI 0.95 ± 0.07; in the EHS 3/4 group (*n*=38), PSV was 22.81 ± 2.79, EDV 3.28 ± 1.02, and RI 0.72 ± 0.05. CDDU, a minimally invasive and accurate method after ICI, is commonly performed in patients with urethral structure and can also determine the relationship between ED and trauma, which is considered as the gold-standard technique for evaluating penile hemodynamics [23]. Therefore, CDDU has been of great significance in classifying the cause and designing the course of treatment for ED, and therefore it is commonly performed in ED patients [24]. During CDDU, the PSV, EDV, and RI were measured. A flow rate of 30 cm/s or higher PSV represented normal arterial flow while a rate of 25 cm/s or lower PSV was considered to be arterial insufficient hence causing ED, and together with a normal arterial response, EDV > 6 cm/s and RI < 0.6 were considered to be normal [8]. In consistency with our findings, Pezeshki Rad et al. [25] demonstrated that both sensitivity and specificity of CDDU in diagnosing traumatic limb vascular injury was much higher, proving that CDDU is a promising tool in screening and diagnosis. Additionally, all patients in the EHS 1/2 group had a single-peak spectrum in systole with no or a rather low spectrum of blood flow in diastole, while patients in the EHS 3/4 group all had an increased blood flow spectrum in both systole and diastole after erection. Furthermore, in comparison with the EHS 1/2 group the proportion of patients with history of hypertension, hyperlipidemia, diabetes, and masturbation in the EHS 3/4 group was relatively low. Previous studies have also provided significant evidence on proving that any history related to testosterone deficiency, hypertension, hyperlipidemia, diabetes, and masturbation was indicative risk factors in ED [26,27].

Most importantly, we found that 4D-CTA was capable of displaying the cavernous artery, internal carotid artery, and penile dorsal artery simultaneously and, was capable of revealing the lesion sites clearly. With comparatively high diagnostic and angiogram accuracy, 4D-CTA using 320-detector row CT would appear as a very practical and useful method in diagnosing arterial diseases [12]. After angiography, a multislice CTA inspection clearly depicted draining veins, tumor feeding arteries, venous sinuses, and even fine branches and vessels near the skull [28]. According to 4D-CTA differentiating arteriovenous fistula subtypes, Beijer et al. [29] suggested that angiography should be considered as the gold standard for acquiring highly detailed image of arterial diseases and 4D-CTA was an additional first-line method for examining patients with arterial diseases. The use of 4D-CTA to diagnose ED yielded AUC of 0.879, with specificity of 93.9%, sensitivity of 82.5%, demonstrated that the accuracy of 4D-CTA diagnosing arterial ED was as accurate as CDDU. 4D-CTA was able to show each stage of contrast passage clearly and the after processing of 4D-CTA was very straightforward by automatic software like MIStar [13]. In addition, with the establishment of a low-dose radiation protocol, 320-detector row dynamic volume CT achieved a relatively low radiation dose at 5.08–5.87 mSv, which ensured a justified level of radiation exposure [30]. Under 320-detector row dynamic volume CT, the entire heart could be covered within one heartbeat [11].

To conclude, the present study demonstrated that 4D-CTA using 320-detector row dynamic volume CT is an extremely promising tool in diagnosing of arterial ED. As the study was conducted with a limited number of patients and a relatively low number of studies concerning 4D-CTA using 320-detector row dynamic volume CT in diagnosing arterial ED, its potential diagnostic accuracy still needs further experimentation and confirmation.

## Abbreviations

4D-CTA: four-dimensional CT angiography
AVSS: audiovisual sexual stimulation
CDDU: color duplex Doppler ultrasound
DSA: digital subtraction angiography
ED: erectile dysfunction
EDV: end diastolic velocity
EHS: erection hardness score
ICI: intracavernous injection
MIP: maximum intensity projection
MPR: multiple planar reformation
NPT: nocturnal penile tumescence
PSV: peak systolic velocity
RI: resistant index
ROC: receiver operating characteristic
SAS: Self-rating Anxiety Scale
SDS: Self-rating Depression Scale
VR: volume rendering
